# Supplementary material for: The Helix-Loop-Helix motif of human EIF3A regulates translation of proliferative cellular mRNAs
Source: PLoS One. 2023 Sep 28;18(9):e0292080. doi: 10.1371/journal.pone.0292080 (PMC10538695; doi:10.1371/journal.pone.0292080)

Figure 1

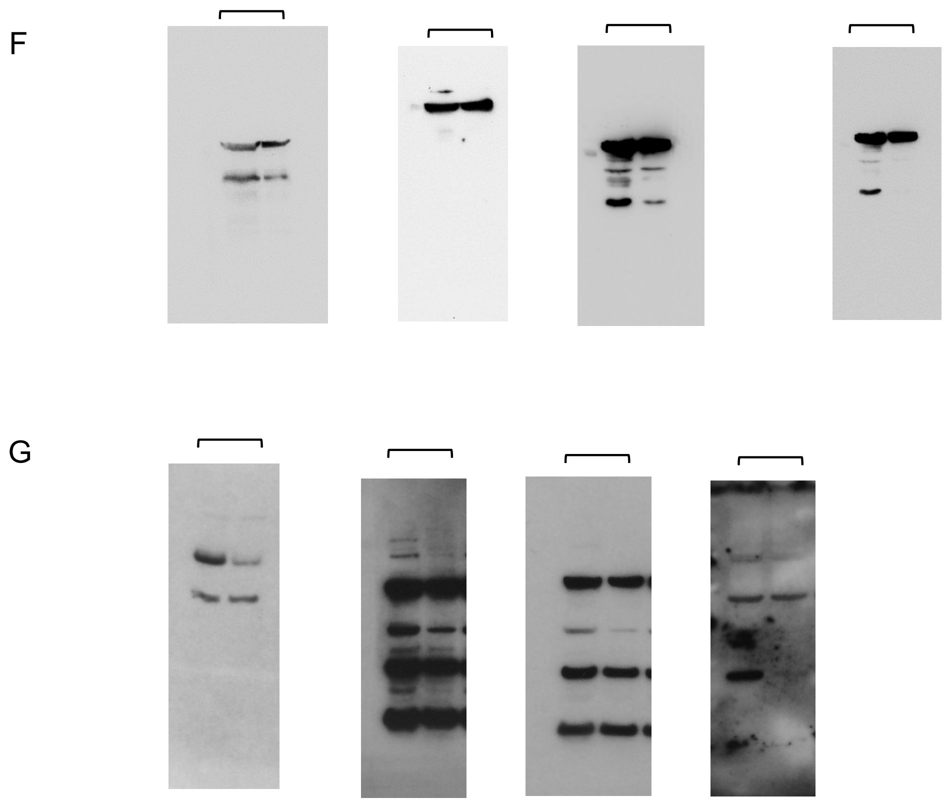

Figure S1

A

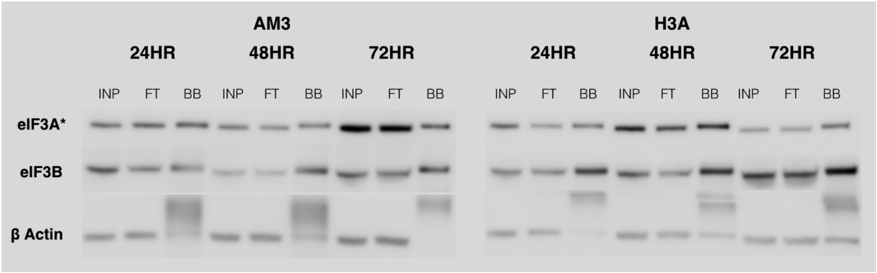
AM3
24HR
48HR
72HR
H3A
24HR
48HR
72HR
INP FT BB INP FT BB INP FT BB
INP FT BB INP FT BB INP FT BB
eIF3A*
eIF3B
β Actin

B

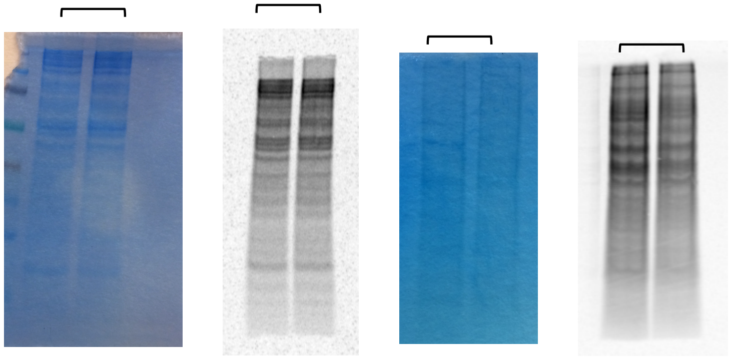

Figure 2

B

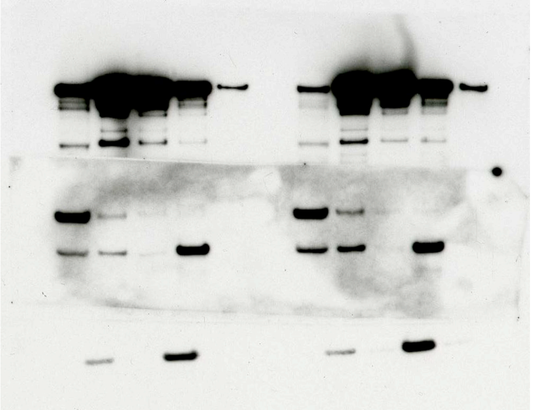

Long exposure

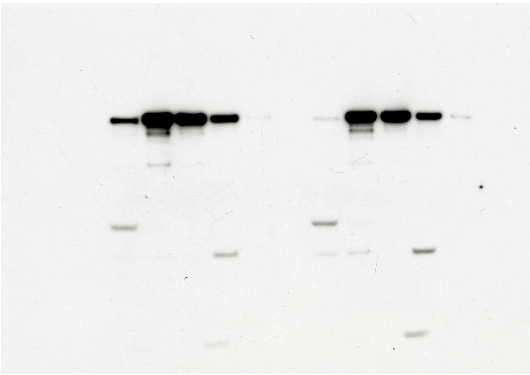

Short exposure

C

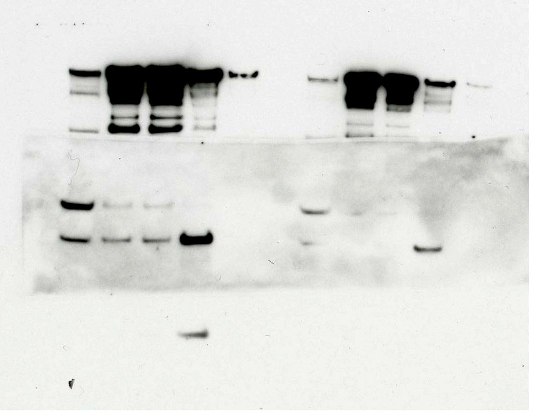

Long exposure

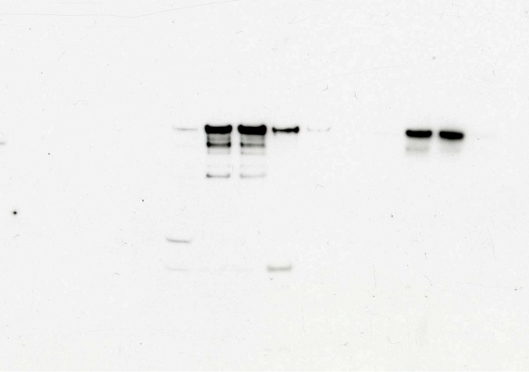

Short exposure

Figure 2 cont.

B

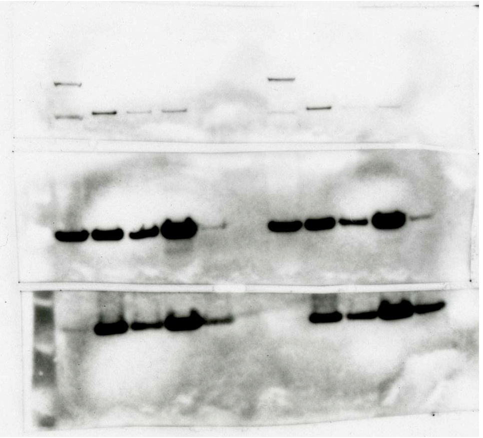

Long exposure

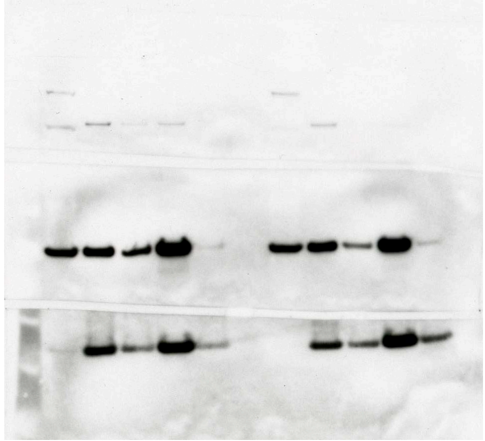

Short exposure

C

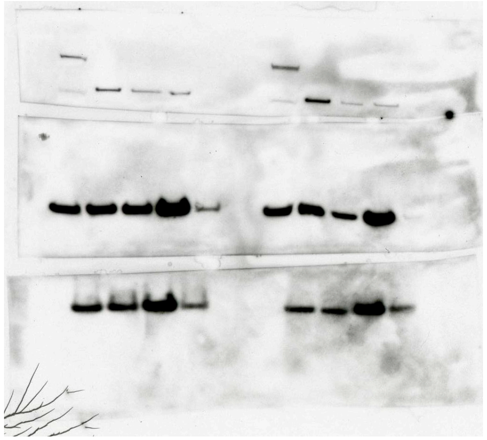

Long exposure

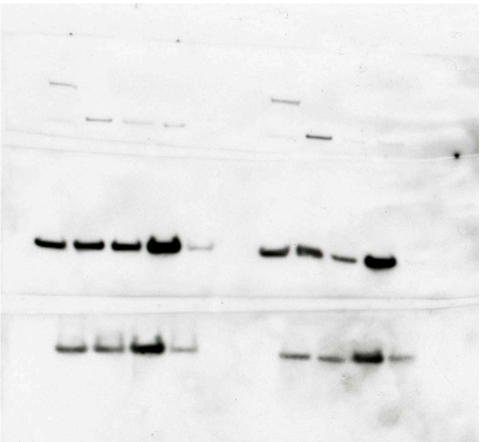

Short exposure

Figure 2, cont.

E

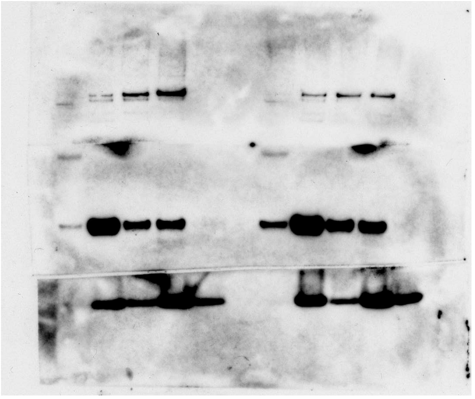

Long exposure

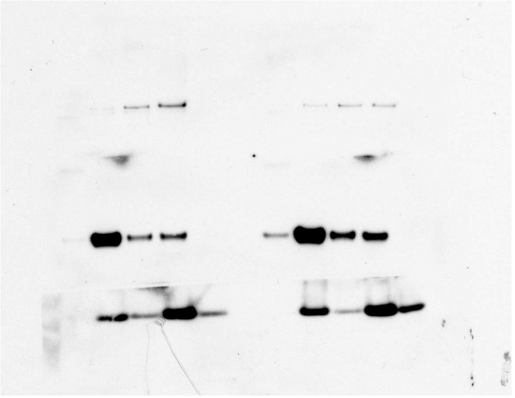

Short exposure

F

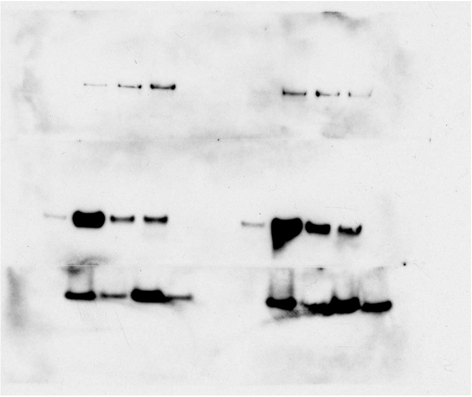

Long exposure

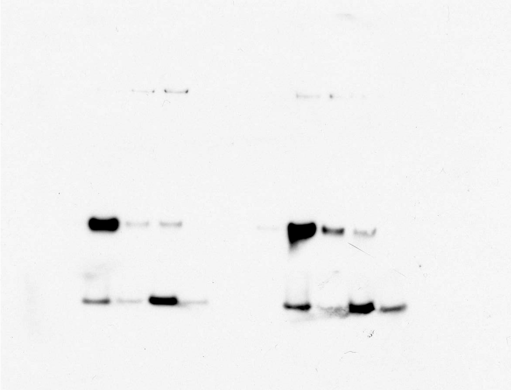

Short exposure

Figure 2, cont.

E

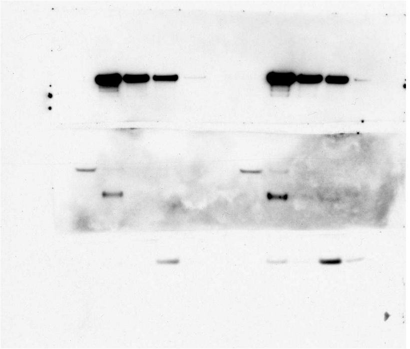

Long exposure

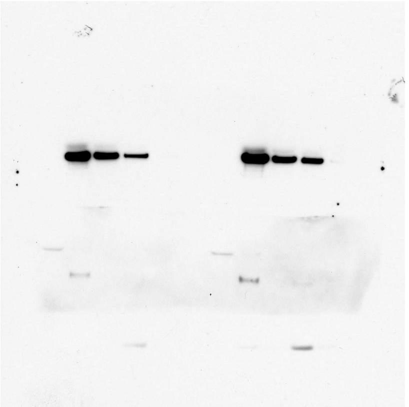

Short exposure

F

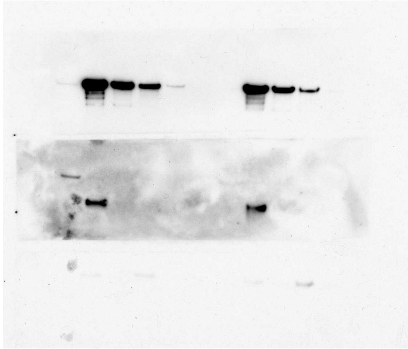

Long exposure

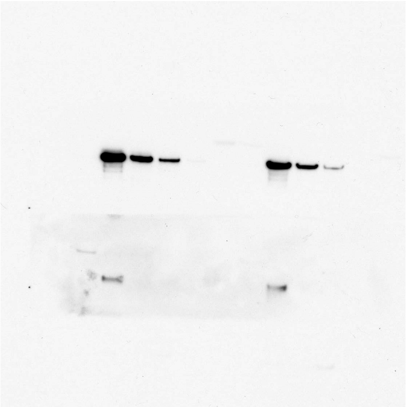

Short exposure

Figure S2

D

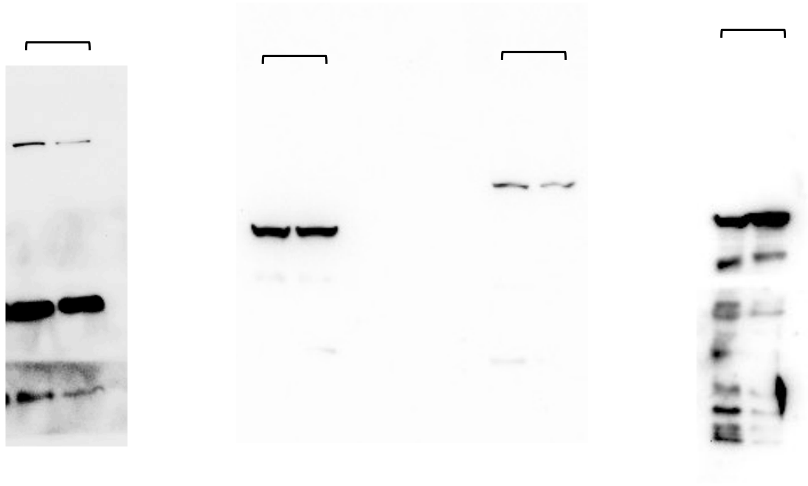

Figure 3, S4C

D

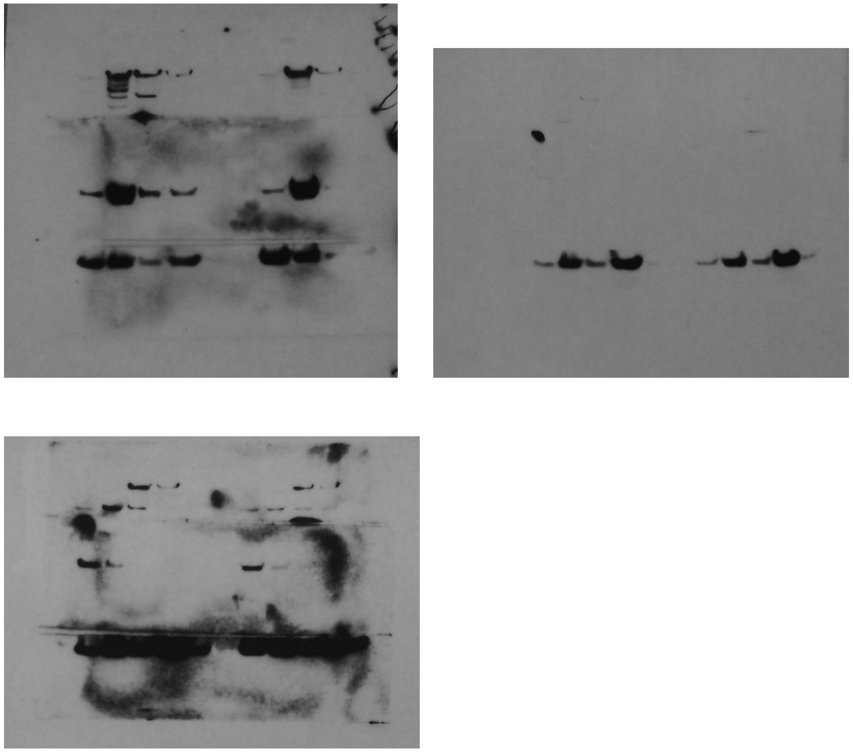

E

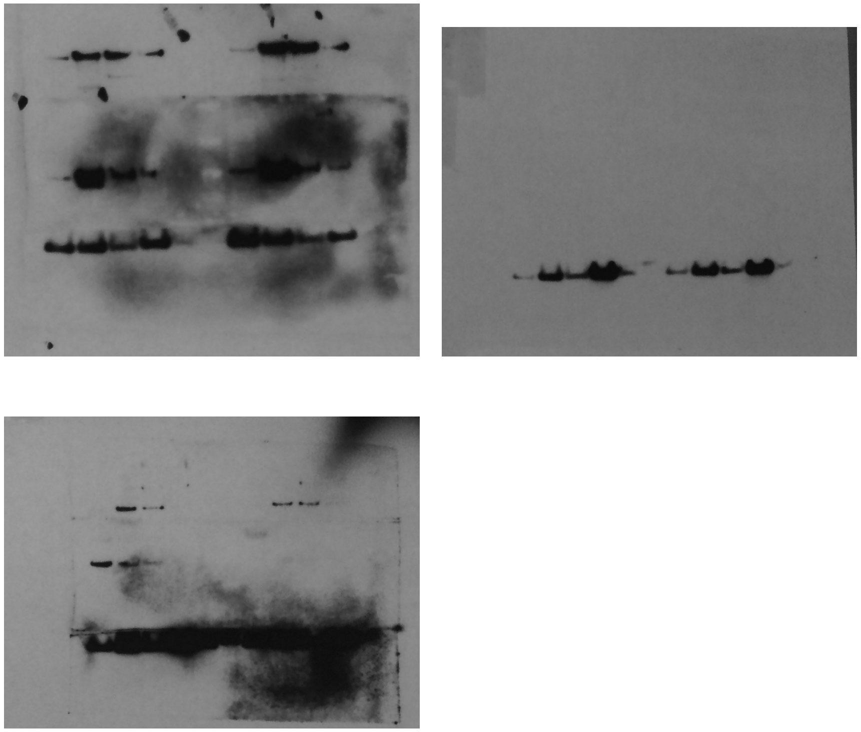

Figure S3

B

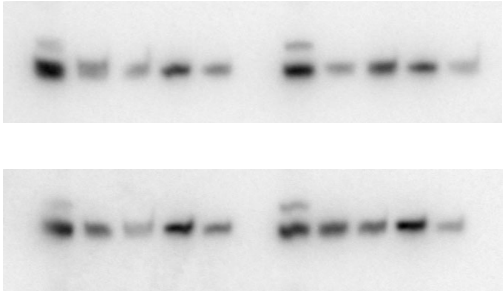

C

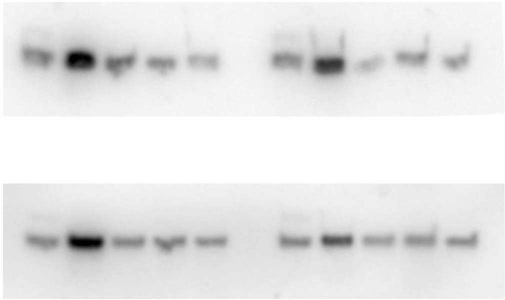

Figure 4, S6A

A

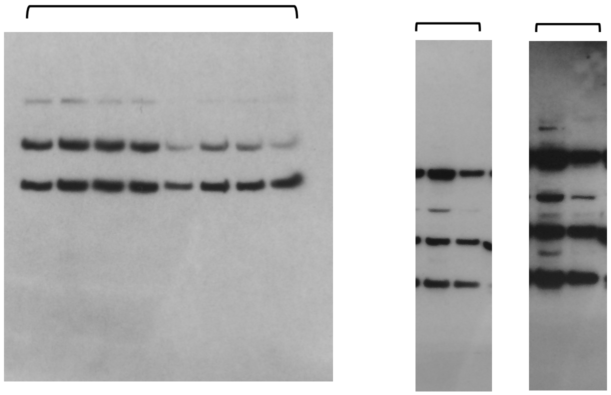

Figure S4, cont.

A

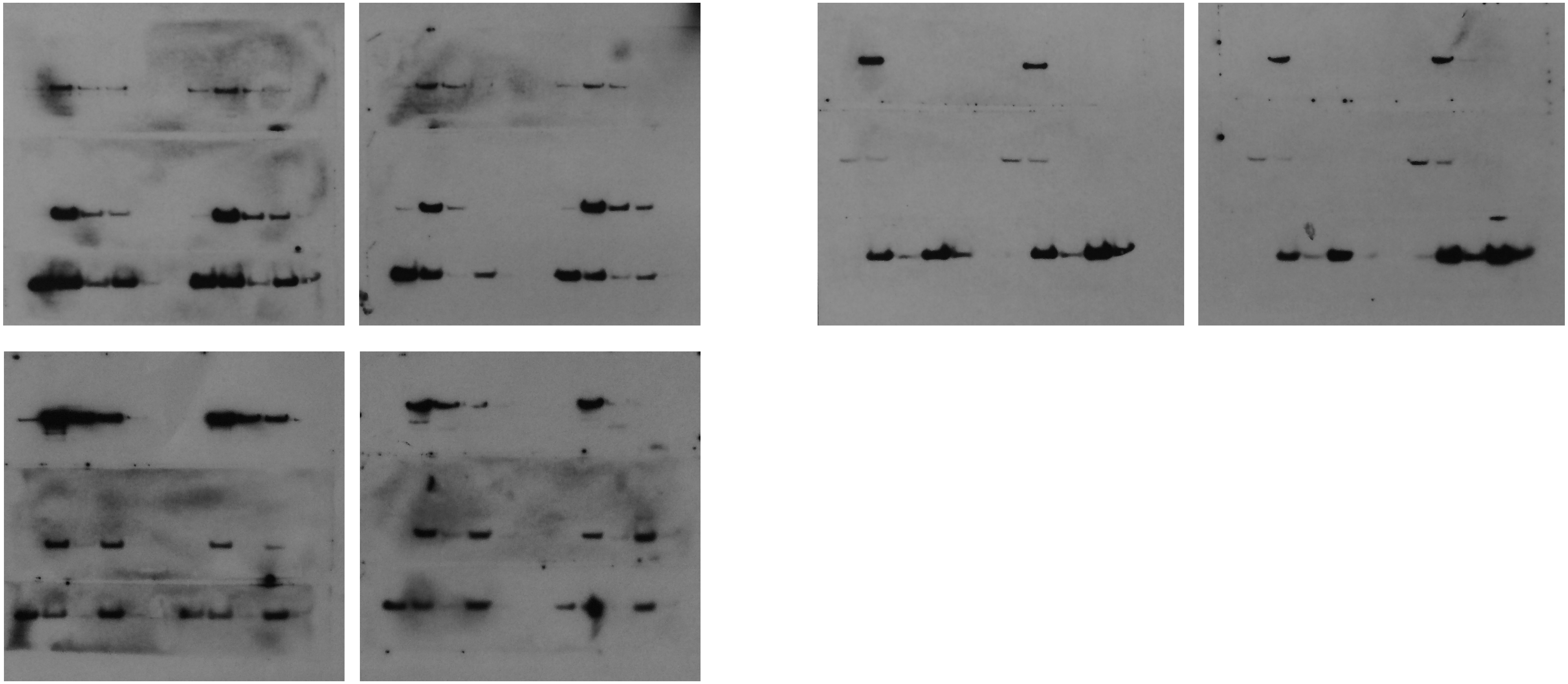

Figure S4, cont.

B

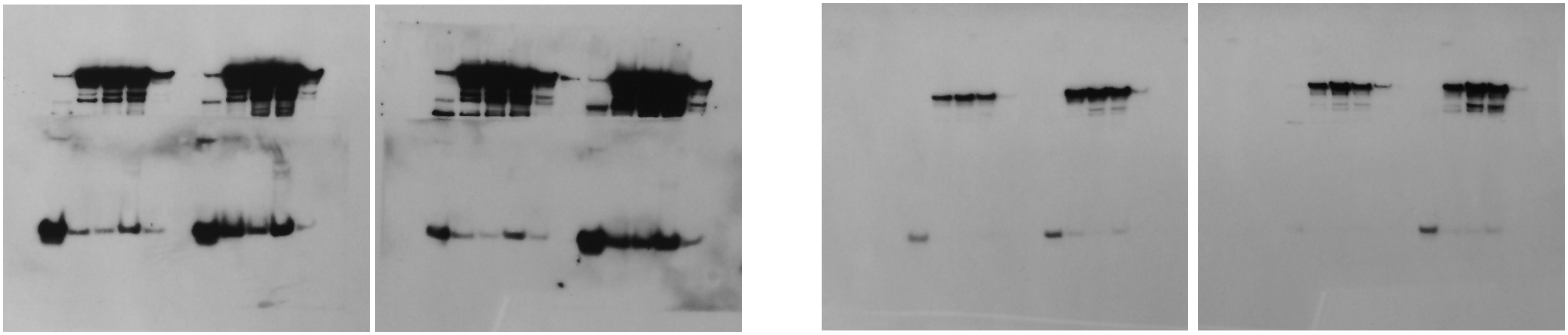

Long exposure

Short exposure

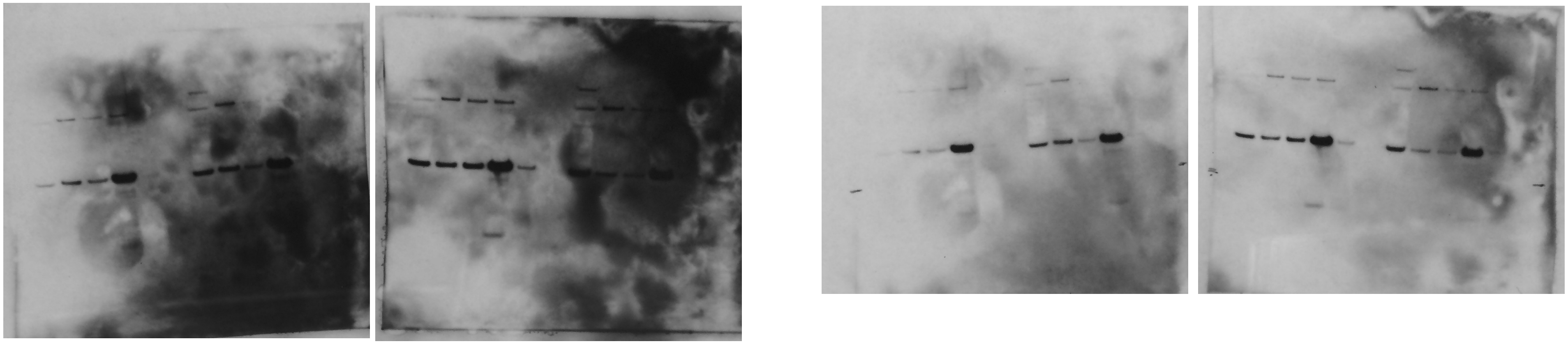

Figure S4, cont.

B

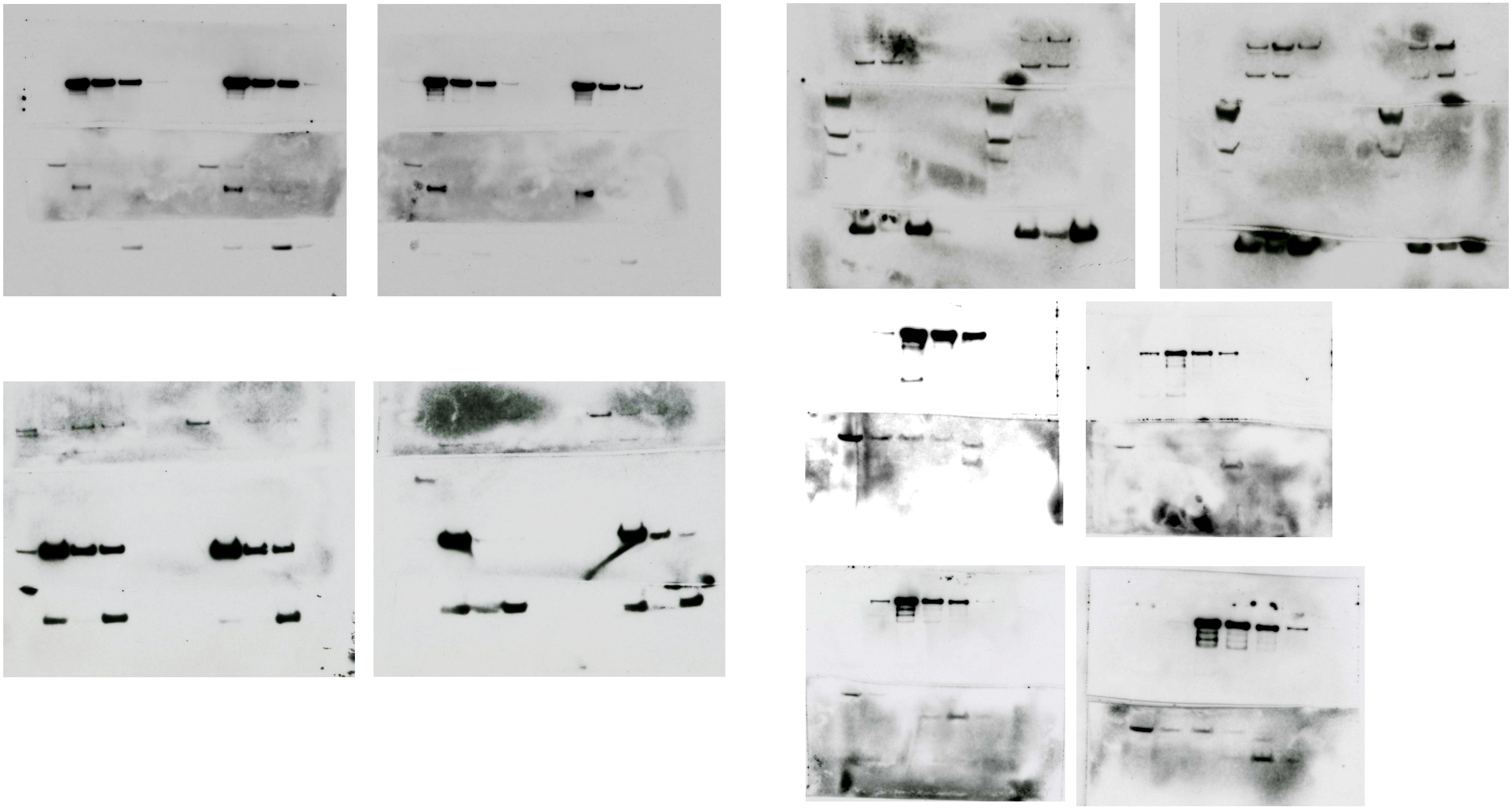

Figure S4, cont.

D

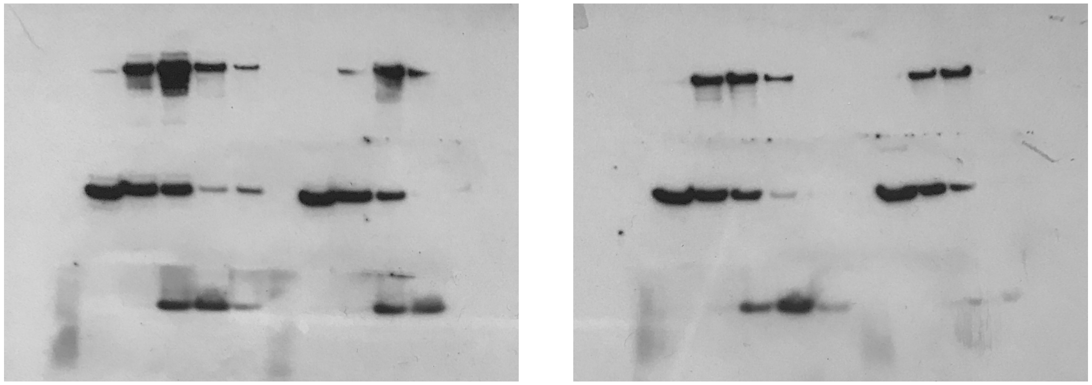

E

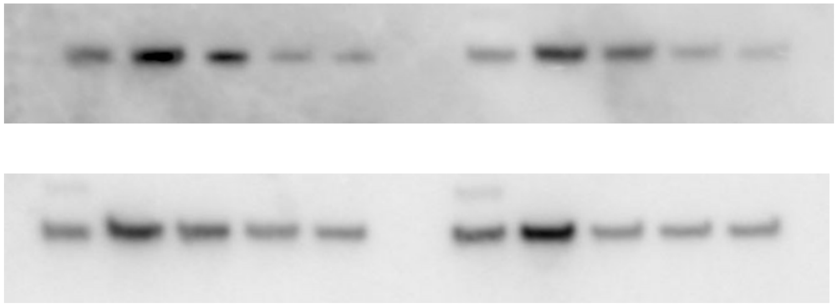

Figure S5

C

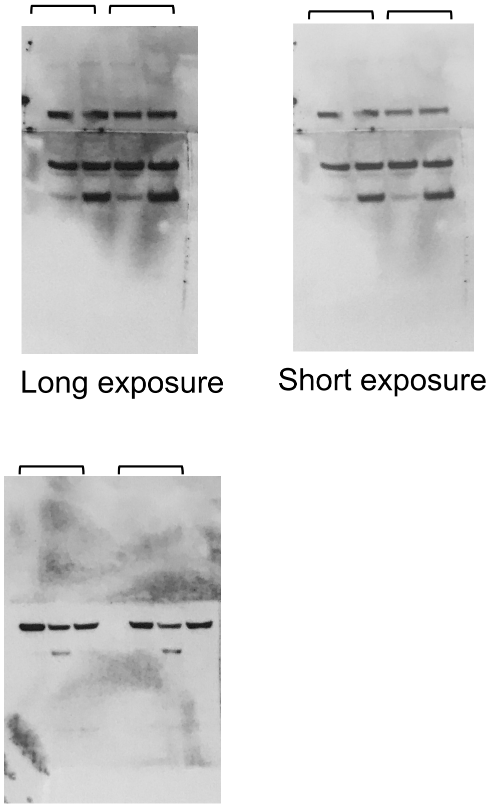

Long exposure

Short exposure

Figure S6

A

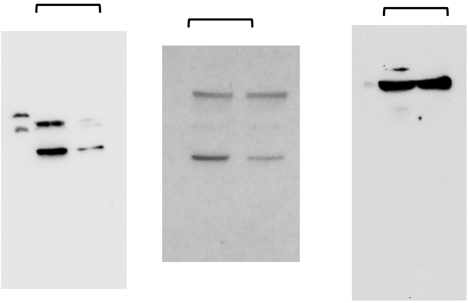

B

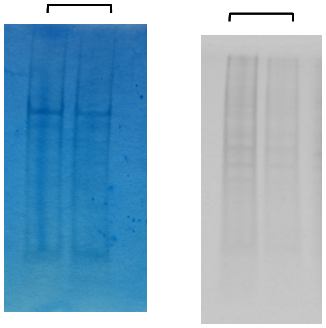

Supplement: S1 Raw images — (PDF) [file pone.0292080.s003.pdf]
