# Supplementary material for: The Helix-Loop-Helix motif of human EIF3A regulates translation of proliferative cellular mRNAs
Source: PLoS One. 2023 Sep 28;18(9):e0292080. doi: 10.1371/journal.pone.0292080 (PMC10538695; doi:10.1371/journal.pone.0292080)
Supplement: Attachment — Submitted filename: Volegova-etal-response-Final.pdf [file pone.0292080.s004.pdf]

5. Review Comments to the Author

Reviewer #1: In this manuscript, the authors report their studies on the helix-loop-helix motif of eIF3a n regulating eIF3a function in translational control of cancer-relevant mRNAs such as Myc. While some findings are interesting and worthy pursuing, it appears that this manuscript was put together with data that do not show cohesiveness and logic. It also lacks rigor for publication.

1. The authors changed silencing methods from shRNA to CRISPRi. However, RNAseq to verify knockdown of endogenous eIF3a by CRISPRi is missing.

We have updated our analysis to show knockdown in the CRISPRi HEK293T and shRNA Ramos cells, using RNASeq data. This information is included in the supplement in Figure S6C. We note that the RNASeq experiment was carried out after 3 weeks of cell passage, during which we observe some reversion of the phenotype, i.e. increased cell proliferation. This effect occurred in all three sets of experiments we carried out (shRNA, CRISPRi in HEK293T cells, shRNA in Burkitt's lymphoma cells), pointing to the toxicity of the HLH* mutation in EIF3A. We have expanded the Discussion to elaborate on this in more depth.

2. Although Figure S1A shows a pulldown of Flag-eIF3a with eIF3b, the pulldown (co-IP) to show interaction between untagged eIF3a and eIF3b, which were used for all subsequent studies is missing.

We thank the reviewer for pointing this out. Co-IP of EIF3A using the anti-EIF3B antibody in this study has been well validated in the published literature, which we now cite in the Methods (references 3 and 54, Lee *et al.* 2015 and De Silva *et al.* 2021).

3. The fractionation and Western blot appear to have been done only once with most western blot shown in low quality including unexplained asterisks on the blot. As more scientific rigor is required by NIH, this quality of work supported by NIH does not meet its standards.

We thank the author for pointing out the need for replicates. We had included replicates in Figures S4A and S4C. Additional replicate blots are now included in Figure S4. We note the asterisk was explained in the figure legend. Also, we now include additional replicate western blots of MET and PRL3 for the HEK293T cell lines in Figure S2D.

4. The rationale in studying the selected eIFs (Fig. 2) is not clearly stated.

We note the basis for the study in the Introduction in paragraphs (1) and (2), where we describe the known interactions between eIF3 subunits and other initiation factors (eIFs) (references 10-11, Sokabe et al. 2012, Eliseev et al. 2018).

5. Sensitization of by RocA is confusing since HLH*eIF3a already released eIF4A1. This study needs discussion and explanation.

We have expanded the Discussion section to cover this point more clearly, with reference to Chu et al. 2020 (reference 44) to explain the nuanced mechanism of action of rocaglates (RocA).

6. Study of ATF4 is also confusing. Firstly, it is not one of the genes found in the sequencing data. Secondly, it is not relevant to the study of Myc regulation or Myc function in the subsequent studies on cancer. Perhaps, similar studies should be performed for Myc but it is unclear if Myc mRNA has uORFs.

Although the MYC experiments are one focus of the study, we deliberately chose ATF4 as an HLH-insensitive mRNA which is highly responsive to stress conditions, as we describe in the text ("EIF3A HLH motif interacts with mRNAs in counterpoint to EIF4A1" section on pages 8-9). As we show in Fig. 3, we assessed whether the combinatorial effect of RocA and the HLH mutation may impact the translation of ATF4 and the ATF4 uORF variants.

7. While the finding that HLH*eIF3a makes RAMO cells proliferate slower and more sensitive to doxorubicin is interesting, this study barely scratched the surface.

We agree this is an interesting direction to pursue in future studies, but we consider the preclinical exploration of the synergy of the HLH mutation with chemotherapeutic agents to lie beyond the scope of this mechanistic study.

It is unclear how reduction of Myc by HLH*eIF3a sensitizes RAMO cells to doxorubicin. Slow growing cells are normally more resistant to drugs such as doxorubicin. The author's finding is also inconsistent with literature where Myc stabilization or upregulation sensitizes cells including RAMO to doxorubicin.

This is an interesting point, and we postulate that the combinatorial effect of transcriptional and translational stress accounts for the sensitization. In regard to MYC protein stabilization, the two situations are not directly comparable. We are decreasing MYC levels due to a translational defect mediated by EIF3A-HLH*, thereby preventing further MYC protein from being synthesized. In the case of cancer cells driven by transcription factors (e.g. Ramos), the stoichiometry of the oncogenic driver (e.g. MYC) is critical to cell survival. Therefore, while increased MYC may sensitize cells to doxorubicin, a deficit of MYC protein can cause equally deleterious effects, as we demonstrate in this study. As noted above, future experiments will be needed to sort out the full implications of our mechanistic results in an *in vivo* system.

Reviewer #2: The manuscript titled "The Helix-Loop-Helix" motif of human EIF3A regulates translation of proliferative cellular mRNAs" is very well written, and thoughtful control experiments are performed. For example, authors used a lentiviral vector to express EIF3F HLH* under hygromycin selection and then depleted endogenous EIF3A using 3'UTR-specific shRNA under dual hygromycin/puromycin selection. Another good example is when the authors used CRISPRi-mediated depletion of endogenous eIF3A and then expressed HLH* using a lentiviral vector. Significant and meaningful ribosome profiling data has been generated which serves as the basis for selecting three different mRNAs to look at. Overall, this is a very nice mechanistic manuscript. However, I have some comments and questions as listed below. Some of these do require further experimentation.

For Fig. 1C: The authors should perform western blot analysis to show the depletion of endogenous eIF3A and expression of FLAG HLH*.

Unfortunately, it is not possible to show depletion of endogenous EIF3A at the protein level, as there are no epitopes in endogenous EIF3A that are not shared with the tagged exogenous version of EIF3A.

The authors have checked the IRES activity in Fig 1B using EMCV. They should also check the IRES activity of myc 5' UTR, as it is very well known that myc expression is regulated by an IRES element.

We thank the reviewer for raising this interesting point. In fact, there is no convincing evidence that MYC expression involves an IRES. The use of an IRES for MYC translation was definitively ruled out by experiments in Andreev *et al.* 2009, which we now cite (ref. 38). This was shown by those authors in multiple ways. It is unfortunate that this paper is not more widely known. We have expanded our discussion to include this point.

Authors showed in their ribosome profiling experiment that they found 149 negatively regulated and 18 positively regulated mRNAs. However, they have not provided a clear rationale as to why they chose three mRNAs (out of 167 mRNAs) to follow up on.

Although it would be interesting to follow up on additional mRNAs, we focused on this set of mRNAs due to their significance to cell proliferation.

In their CRISPRi-mediated depletion of endogenous eIF3A and subsequent exogenous HLH* expression using lentiviral vector experiment the authors should show the level of eIF3A (endogenous) and HLH* by western blotting (PDF page # 12).

We thank the reviewer for this suggestion, but as noted above, it is not possible to distinguish the endogenous WT and exogenous HLH* EIF3A by western blot, as they have the same epitopes. We note that in Fig. S6B that global protein synthesis is repressed slightly in the Burkitt's lymphoma cells in the presence of the exogenous HLH* EIF3A but not with exogenous HLH (WT) EIF3A. Also, as noted for the first comment by Reviewer 1, we have now analyzed RNA-Seq data from the CRISPRi Burkitt's lymphoma cells, where we show approximately equal levels of endogenous and HLH* EIF3A mRNA levels after 3 weeks of passage. At this point the cells begin to show a phenotypic reversion, i.e. increased cell proliferation.

After nuclease treatment of their cell lysates, the authors need to confirm that the nuclease activity has been effectively terminated by EGTA treatment. I see that they have used EGTA but then did not confirm that the nuclease activity has been stopped.

The use of nuclease treatment to remove endogenous mRNAs followed by EGTA treatment is common practice in the field, and we have followed well-established protocols in these experiments. See for example Lee *et al.* 2015, ref. 3.

In the ribosome fractionation experiments, the authors have not shown the presence of GAPDH or MYC mRNAs presence in any of their fractions (48S and 80S). This should be done otherwise it becomes difficult to imagine that ribosome is actually recruited on these mRNA, particularly when the authors did not show the effectiveness of EGTA treatment of their lysates.

As noted above, we have followed well-established protocols for lysate preparation, including nuclease treatment and EGTA inactivation of the nuclease.

In the same figure (Fig 2) the authors should perform fractionation of their control lysate WITHOUT priming with GAPDH or MYC mRNAs.

The use of extracts depleted of cellular mRNAs is widespread in the field, as noted above, and we think our experiments are in line with established methods.

Are both GAPDH and MYC mRNA capped? If so, the authors should perform this experiment with A-capped myc mRNA.

As noted above, the *MYC* mRNA does not use an IRES mechanism. The A-cap was used in Andreev et al. 2009 (ref. 38), in which the MYC IRES was ruled out. Please see Fig. 4B in that paper.

On PDF page # 12, (manuscript page 6), the authors claim that they have observed a 'defect' in eIF3A and eIF5B distribution …. On what basis? Amount of these proteins detected by western blots? Band density? If so, they have not normalized them to loading control.

As noted in the Figure 2 legend and the Methods ("Sucrose gradient fractionation" section), we normalized protein loading by quantification of total protein in the concentrated sucrose gradient fractions and by including the RPS19 loading control to demarcate the localization and amount of the 40S ribosomal subunit between different gradients.

The authors did not show the interaction of eIF3A, and the lack of HLH*, with MYC mRNA. They should show this by performing REMSA or filter binding assays.

We thank the reviewer for this idea, but we do not think EIF3A on its own is likely to bind to the mRNA, without other translation factors and the 40S subunit being involved. We note more clearly in the Discussion that we expect the EIF3A mutation likely weakens contacts to the 40S subunit, and this defect would disrupt favorable interactions with select mRNAs in the context of preinitiation complexes.

The authors have used only one Ramos Burkitt's Lymphoma cell line, but what if the effect they are seeing is cell line specific? They need to perform the cell viability assays in at least three cell lines.

We think this kind of experiment could be done in future studies of eIF3A-HLH*, but we think this request is beyond the scope of the present manuscript. We now note in the Discussion that how general the effect of eIF3A-HLH* will be in other MYC-addicted cancers will need to be explored in the future.

Other Figure-specific comments:
They should use the standard error of mean and t-test in all their biological replications and not the standard deviation of biological triplicates.

We note that we have applied t-tests for the bar graphs previously but have modified our figure legends for clarity. For the bar graphs and growth curves, we think reporting standard deviations is more appropriate to convey the variability in the measurements, given the small sample sizes (triplicates). We apologize for the confusion.

The molecular wt mentioned for eIF5B in Fig 2 seems to be inaccurate.

We thank the reviewer for the comment, and we have doubled-checked the MW. The mass of eIF5B of eIF5B is 139 kDa (see https://www.uniprot.org/uniprotkb/O60841), which is labeled in Fig. 2 to be observed between 100 and 150 kD.

uORF2 should overlap with the CDS for ATF4 gene organization figure (Fig 3F). I have checked their mRNA sequence in the supplementary file, which seems to be correct.
However, the presentation of the ATF4 organization in Fig 3F is incorrect.

We thank the reviewer for noticing this. We have modified the figure accordingly.

Important: in Fig 4 the authors claim that HLH* mutation sensitizes Burkitt's lymphoma cells through loss of MYC. However, they have not established a clear link for the effect on cell viability. Yes, they do see significant cell death in HLH* with doxorubicin treatment. However, this cannot be only attributed to the loss of MYC. In order to claim that the authors should perform an experiment where they exogenously express MYC in HLH* mutant background and perform the same visibility assay. The reversal of cell death in the suggested condition would suggest the sensitization to doxorubicin was actually through MYC.

We thank the reviewer for this suggestion. It is correct that MYC may not be the sole cause of the effect, since we have introduced a translation defect that could affect other mRNAs. We have modified the Discussion accordingly.

Minor points:
PDF page # 9, it should be in contrast with not "By contrast with…"
Fig S1: did they perform transient transfection of lentivirus or transduced lentivirus?

As noted in the Methods we used transduced lentiviruses.

Reviewer #3: The manuscript by Volegova et al. investigated the role of the Helix-Loop-Helix (HLH) RNA binding motif of EIF3A in regulating translation of cellular mRNAs. The HLH motif was previously known to aid IRES-mediated translation of Hepatitis C Virus. In this study, the authors reported that the HLH motif regulates the translation of a subset of oncogenic mRNAs, notably MYC. Using HLH mutant cells, the authors showed that the translation of MYC, MET and PRL3 is reduced. Mechanically, they proposed that the HLH motif modulates the interaction between eIF3A and EIF5B, and also affects the interaction between EIF4A1 and 43S pre-initiation complex (PIC).

By focusing on the role for the HLH motif of EIF3A, it is interesting to find that translation of a small subset of genes is affected by the EIF3A mutant. The proposed mechanism differs from IRES, although it's unclear how the HLH domain of EIF3A modulates the helicase EIF4A1 in the scanning process. Overall, the manuscript was well-written and the results were nicely presented. The manuscript would be improved if the following concerns are addressed.

Major concerns:
1. Figure 2, it would be nice to show the sucrose gradient profiles in the presence of wild type or HLH mutants. This is important to demonstrate the overall translation capacity.

We thank the reviewer for this suggestion. We have added these to the plots.

2. Similarly, what does the polysome profile look like in CRISPRi-edited cell lines?
In fact, we carried out these experiments with the CRIPSRi cell lysates. We have clarified this in the Methods (see the "*In vitro* translation" and "Sucrose gradient fractionation" sections).

3. Figure 4, the protein level of ACTB are greatly reduced in HLH RAMOS cells. Is the presence

of HLH mutant toxic to the cell? Besides cancer cells, is the presence of HLH mutant toxic to normal cells as well?

We thank the reviewer for this comment. As noted above, we have changed the Discussion to note the possible effects of the eIF3A-HLH* mutation in RAMOS cells and refer to Figure. S6B, which shows an overall reduced level of translation.
