# Supplementary material for: The Helix-Loop-Helix motif of human EIF3A regulates translation of proliferative cellular mRNAs
Source: PLoS One. 2023 Sep 28;18(9):e0292080. doi: 10.1371/journal.pone.0292080 (PMC10538695; doi:10.1371/journal.pone.0292080)
Supplement: Attachment — Submitted filename: Volegova-etal-2ndesponse-Final.pdf [file pone.0292080.s005.pdf]

6. Review Comments to the Author

Reviewer #1: In response to the comment on the inconsistent observation of RAMO cell response to doxorubicin due to Myc down-regulation from literature, the authors provided an argument on different mechanism may be used. Because this is an important issue and it likely will create controversies, the authors are advised to provide an extensive and balanced discussion on this issue, perhaps including discussion not only on Myc but also of eIF3a in drug response.

As suggested, we have added a short section to the final paragraph of the Discussion noting the conflicting results in the literature (new refs. 46-48). This addition fits well with the following statements that highlight the need for future experiments to better understand EIF3A and MYC roles in different cancer contexts.

Reviewer #3: In this revised manuscript, the authors have addressed the main concerns. As a result, the manuscript is suitable for publication.

We thank the reviewer for the positive feedback.
